# Acute and overuse injuries among sports club members and non-members: the Finnish Health Promoting Sports Club (FHPSC) study

**DOI:** 10.1186/s12891-019-2417-3

**Published:** 2019-01-19

**Authors:** L. Ristolainen, K. Toivo, J. Parkkari, S. Kokko, L. Alanko, O. J. Heinonen, R. Korpelainen, K. Savonen, H. Selänne, T. Vasankari, L. Kannas, J. Villberg, U. M. Kujala

**Affiliations:** 1 Orton Orthopaedic Hospital, Orton, Helsinki, Finland; 2 Tampere Research Center of Sports Medicine, Tampere, Finland; 3 Faculty of Sport and Health Sciences, University of Jyväskylä, Jyväskylä, Finland; 4 Sports Medicine Clinic, Foundation for Sports and Exercise Clinic, Helsinki, Finland; 5 Paavo Nurmi Centre & Department of Physical Activity and Health, University of Turku, Turku, Finland; 6 Oulu Deaconess Institute, Department of Sports and Exercise Medicine, Oulu, Finland; 7 Medical Research Center Oulu Oulu University Hospital and University of Oulu, Oulu, Finland; 8 Center for Life Course Health Research, University of Oulu, Oulu, Finland; 9 Kuopio Research Institute of Exercise Medicine, Kuopio, Finland; 10 Department of Clinical Physiology and Nuclear Medicine, Kuopio University Hospital, Kuopio, Finland; 11 Department of Psychology, University of Jyväskylä, Jyväskylä, Finland; 12 UKK Institute of Health Promotion Research, Tampere, Finland

**Keywords:** Athletic injury, Acute injury, Overuse injury, Adolescent, Sports club member, Non-member

## Abstract

**Background:**

Physical activity in adolescence is promoted for its multi-dimensional health benefits. However, too intensive sports participation is associated with an increased injury risk. Our aim was to compare the occurrence of acute and overuse injuries in Finnish sports club members and non-members and to report training and competing habits associated with a higher injury risk in sports club members.

**Methods:**

In this cross-sectional survey targeted at 14–16-year-old adolescents, a structured questionnaire was completed by 1077 sports club members and 812 non-members. The main outcome measures were self-reported acute and overuse injuries, their location and type.

**Results:**

At least one acute injury in the past year was reported by 44.0% of sports club members and 19.8% of non-members (*P* < 0.001). The sex-adjusted odds ratio (OR) for acute injury in sports club members compared to non-members was 3.13 (95% confidence interval (95% CI) 2.54–3.87). Thirty-five percent of sports club members and 17.4% of non-members (*P* < 0.001) reported at least one overuse injury during the past year. The overuse injury OR for sports club members was 2.61 (95% CI 2.09–3.26). Sports club members who trained 7–14 h per week during training (OR 1.61, 95% CI 1.21–2.12, *P* = 0.001) or competition season (OR 1.55, 95% CI 1.18–2.06, *P* = 0.002) were more likely to report an injury compared to members who trained 3–6 h per week. Those sports club members who participated in forty competitions or more compared to 7–19 competitions per year were more likely to report an acute injury (OR 1.55, 95% CI 1.05–2.08, *P* = 0.028) or for an overuse injury (OR 1.53, 95% CI 1.02–2.30, *P* = 0.038).

**Conclusions:**

Both acute and overuse injuries are common among youth sports club members, and the number increases along with increasing amounts of training and competitions. More effective injury prevention is needed both for adolescents engaging in sports club activities and for other adolescents.

**Electronic supplementary material:**

The online version of this article (10.1186/s12891-019-2417-3) contains supplementary material, which is available to authorized users.

## Background

Physical activity in adolescence provides multidimensional health benefits [1, 2]. However, the risk of sports injuries increases along with the increasing volume, intensity, and competitiveness of the sports activities [3]. Youth team sports carry a higher injury risk than individual sports [4]. Some earlier studies have focused only on acute [5] or overuse injuries [6] only on either sex [7, 8] or on a specific injury, such as anterior cruciate ligament rupture [9, 10]. There are scattered studies on adolescents covering various different sports and different types of injuries. The occurrence and risk factors of different types of unintentional injuries among Finnish adolescents have previously been studied [11]. The sports injuries of adolescent sports club members have been studied previously [12–14], but studies comparing injuries between sports club members and non-members are rare [13]. Furthermore, the occurrence of overuse injuries has been studied less than that of acute injuries. The American Medical Society for Sports Medicine has made a position statement concerning overuse injuries and burnout, which are common problems in youth sports [15].

Sports clubs are a common way to participate in organized sports in Finland. Nearly half of the adolescent population reported to participate in organized sports in year 2013 [16] and according to the newest report this has raised to as much as 62% [17]. This cross-sectional study is a part of the Finnish Health Promoting Sports Club (FHPSC) study [18]. The purpose of this report is to compare the occurrence of self-reported acute and overuse injuries in Finnish adolescent sports club members and non-members. We also compared the anatomic locations of acute and overuse injuries and the injury types and studied the association between training and competing habits with the risk of injury. The injury definitions used in the questionnaire were obtained from an earlier Finnish study [19]. An acute injury was defined as an injury occurring suddenly keeping one away from exercise for at least one day or requiring a physician’s care [20, 21]. An overuse injury was defined as an injury that causes gradually increasing pain during exercise loading without any noticeable external cause possibly stopping exercise completely [20, 22].

## Methods

### The FHPSC study

The aim of the FHPSC study was to survey health promotion orientation and activity of youth sports clubs and coaches. In addition to surveys, a clinical health examination was performed and the health behavior and health status of youth participating in sports clubs was compared to their non-participating peers.

#### Data collection

The FHPSC data were collected during 2013 in two rounds, from January to May for winter sports, and from August to December for summer sports. The comparison data for non-members were collected accordingly in two rounds via schools (9th grade, 14–16-year-old adolescents). For more details, see Kokko et al. [18].

### Participants

#### The sports club sample

**Fig. 1:**
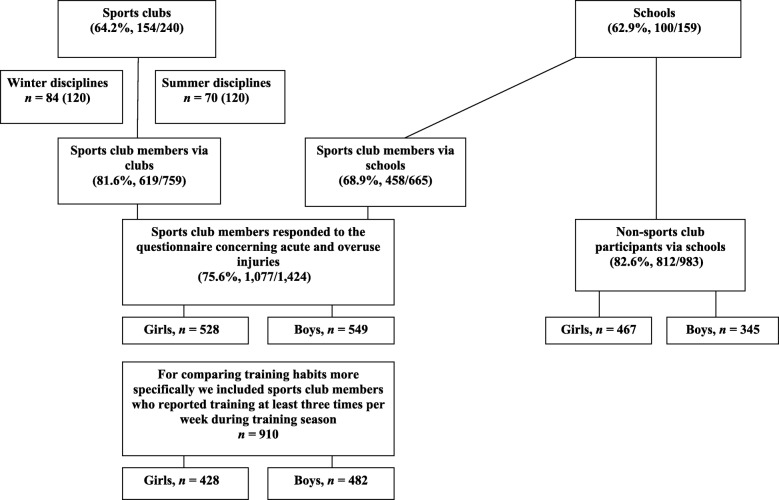
Data collection and the final study group

To obtain a nationally representative sample a total of 240 youth sports clubs from the ten most popular sports disciplines for youth were targeted. Sports clubs were stratified by the following criteria: 1) winter and summer sports, and 2) team and individual sports. Thereafter, the most popular disciplines (basketball, cross-country skiing, floorball, gymnastics, ice hockey, orienteering, skating, soccer, swimming, and track and field) were chosen. The main sports of the sports club members are shown in Additional file 1: Table S1. In total, 1077 youth sports club members (a participation rate of 64%) were included (Fig. 1).

#### The school-based sample

To compare the acute and overuse injuries of youths participating in organized sports clubs (i.e., members) with their non-participating counterparts (i.e., non-members) separate data were collected via schools. These data were also collected in two rounds (winter and summer). Overall, 159 schools were contacted and 100 participated (63%). Members of the school-based sample (*n* = 1648) were asked about their sports club participation, and sports club members in the school data (*n* = 458) were treated as sports club members in the following analysis. The final study sample included 812 non-members and 1077 sports club members (Fig. 1).

### Surveys

Two surveys were conducted for all participants. The first survey focused on the health behaviors, including self-evaluated physical activity. The second survey focused on the adolescents’ injuries and musculoskeletal health, and these questions were identical for both groups. The questions used in the surveys were compiled from previously validated questionnaires (Additional file 2: Table S2) [19, 23–26].

In the injury questionnaire the participants reported the number of acute and overuse injuries they had suffered within the preceding twelve months, the location of the injury, the type of injury, and the circumstances under which they occurred. Additional questions concerning the main sport and training habits were directed to sports club members. Participants who responded to the injury-questionnaire (*n* = 1797, 95%) also responded to a questionnaire on leisure-time physical activity [18]. For more details of the structured questionnaire, see Additional file 2: Table S2. We used the question “How many hours of vigorous exercise/physical activity do you usually do per week in your leisure time (after school lessons)” for both groups to assess the amount of physical activity in order to compare the effect of the physical activity level on the acute or overuse injury risk [27].

“The term “acute injury” was defined as an acute sports injury which occurs suddenly or accidentally interrupting exercise or causing an identifiable trauma, and keeps one away from exercise for at least one day, or requires a physician’s care. The term “overuse injury” was defined as an injury that causes increasing pain during or after exercise without any noticeable external cause of injury and possibly even completely prevents exercise” [19–22].

The study is carried out in conformance with the declaration of Helsinki. A positive statement from the Ethics Committee of Health Care District of Central Finland was received (record number 23 U/2012). All sports clubs participated free-willingly to the study. This was secured by requesting clubs permission at the beginning of study. Thereafter, all adult respondents were notified that they had a right to refuse to participate and withdraw from the study at any time.

### Statistical analysis

Differences between sports club members and non-members were assessed using crosstabs and the chi-squared test and *t*-test when appropriate. Logistic regression was applied to study the associations between injuries (acute and overuse) and sports club membership and sex. Odds ratios (ORs) and their 95% CIs (95% Confidence Intervals) were calculated for the occurrence of acute and overuse injuries, anatomic location, and type of injuries in sports club members compared to non-members and the logistic regression analysis was adjusted for sex. Logistic regression analysis was also applied to study the associations between the injuries and the characteristics of training and competing among the sports club members. Odds ratios and their 95% CIs were also calculated for the occurrence of acute and overuse injury (at least one injury) by sports club participation and by volume of reported leisure-time physical activity, the logistic regression analysis was adjusted for sex. We also more specifically compared the occurrence of acute and overuse injuries and their anatomic location between sports club members training three times per week or more in the training season (*n* = 910) and non-members (*n* = 812). The statistically significant threshold was accepted at *P* ≤ 0.05 (two-tailed). IBM SPSS Statistics (version 22.0) was used to carry out all analyses except the total number of acute and overuse injuries and injury rates in different anatomical locations.

## Results

Fifty-one percent of sports club members (549 of 1077) and 42.5% (345 of 812) of non-members were boys (*P* < 0.001; Fig. 1). Eighty-four percent (*n* = 910: 428 boys and 482 girls) of sports club members reported training at least three times weekly during the training season in the past year.

### Occurrence of acute and overuse injuries

The total number of reported acute injuries in adolescents within the past 12 months was 2478 (Table 1). Forty-four percent of sports club members and 19.8% of non-members (*P* < 0.001) reported at least one acute injury in the past twelve months (Table 1). Boys were more likely to report acute injuries than girls (club membership-adjusted OR 1.31, 95% CI 1.08–1.59, *P =* 0.006). Among sports club members, individual sports’ participants were less likely to report an acute injury than team and contact sports participants (OR 0.59, 95% CI 0.37–0.65, *P <* 0.001). In the sub-group of sports club members training at least three times weekly during the training season, compared to non-members, the sex adjusted OR for reporting at least one acute injury was 3.49 (95% CI 2.81–4.34, *P <* 0.001).

**Table 1:** At least one acute, one overuse injury or at least one acute or overuse injury among sports club members and non-members

	Sports club member				Non-member				
	All	Boys	Girls		All	Boys	Girls		
	*n =* 1077	*n* = 549	*n* = 528		*n* = 812	*n* = 345	*n* = 467		
	*n* (%)	*n* (%)	*n* (%)	*P* Value^*^	*n* (%)	*n* (%)	*n* (%)	*P* Value^*^	*P* Value^#^
At least one acute injury	474 (44.0)	256 (46.6)	218 (41.3)	0.077	161 (19.8)	73 (21.2)	88 (18.8)	0.413	< 0.001
At least one overuse injury	378 (35.1)	188 (34.2)	190 (36.0)	0.550	141 (17.4)	51 (14.8)	90 (19.3)	0.095	< 0.001
At least one acute or overuse injury	649 (60.3)	339 (61.7)	310 (58.7)	0.339	250 (30.8)	105(30.4)	145 (31)	0.851	< 0.001

^*^P Values for statistical difference for the occurrence of injuries between boys and girls among sports club members and non-members

^#^*P* Values for statistical difference for the occurrence of injuries between all sports club members and non-members derived from logistic regression analysis adjusted for sex

The total number of reported overuse injuries within the past 12 months was 1646. Thirty-five percent of sports club members and 17.4% of non-members (*P <* 0.001) reported at least one overuse injury during the past twelve months (Table 1). The difference between boys and girls was not statistically significant for reporting at least one overuse injury. Compared to the non-members, among the sub-group of sports club members training at least three times weekly during the training season, the sex-adjusted OR for reporting at least one overuse injury was 2.84 (95% CI 2.26–3.56, *P <* 0.001).

Sixty percent of sports club members and 30.8% of non-members reported either an acute or an overuse injury during past year (sex-adjusted *P <* 0.001). For more details, see Table 1.

### Injuries by anatomic location

Out of the 1790 acute injuries reported by sports club members the most (13.6%) were injuries of the wrist or hand. This was also the most common acute injury location for non-members (15.1%). The second most common location was the ankle for sports club members and knee for non-members (11.7 and 11.0% respectively) (Table 2). The third most common location for an acute injury in both groups was the foot, 11.5% of all acute injuries for sports club members and 9.9% for non-members were injuries of the foot (Table 2).

**Table 2:** The total number of acute and overuse injuries and percentages (%) in different anatomical locations among sports club members and non-members

	Sports club members (*n* = 1.077)			Non-members (*n* = 812)			Sports club members (*n* = 1.077)			Non-members (*n* = 812)		
	Number of acute injuries						Number of overuse injuries					
	*n*	%*	%**	*n*	%*	%**	*n*	%*	%**	*n*	%*	%**
Head	49	4.5	2.7	20	2.5	2.9						
Face. teeth. Eye area	79	7.3	4.4	32	3.9	4.7						
Shoulder. upper arm. Clavicle	121	11.2	6.8	44	5.4	6.4	46	4.3	4.3	32	3.9	5.5
Elbow. forearm	58	5.4	3.2	29	3.6	4.2	33	3.1	3.1	21	2.6	3.6
Wrist and hand	244	22.7	13.6	104	12.8	15.1	106	9.8	10.0	75	9.2	12.8
Neck. neck region	66	6.1	3.7	35	4.3	5.1	46	4.3	4.3	38	4.7	6.5
Upper back	35	3.2	2.0	19	2.3	2.8	29	2.7	2.7	20	2.5	3.4
Low back	85	7.9	4.7	38	4.7	5.5	94	8.7	8.9	42	5.2	7.2
Chest	33	3.1	1.8	19	2.3	2.8	18	1.7	1.7	12	1.5	2.0
Abdomen	29	2.7	1.6	17	2.1	2.5	15	1.4	1.4	13	1.6	2.2
Hip. groin. Gluteals. pelvis	182	16.9	10.2	53	6.5	7.7	118	11.0	11.1	61	7.5	10.4
Thigh	109	10.1	6.1	32	3.9	4.7	80	7.4	7.6	37	4.6	6.3
Knee	170	15.8	9.5	76	9.4	11.0	178	16.5	16.8	65	8.0	11.1
Calf and shin	79	7.3	4.4	30	3.7	4.4	94	8.7	8.9	38	4.7	6.5
Ankle	210	19.5	11.7	57	7.0	8.3	70	6.5	6.6	46	5.7	7.8
Achilles tendon	35	3.2	2.0	15	1.8	2.2	28	2.6	2.6	18	2.2	3.1
Foot	206	19.1	11.5	68	8.4	9.9	104	7.8	9.8	69	8.5	11.8
All injuries	1790			688			1059			587		

* percentage of sports club members and non-members having sustained an injury in this anatomical area ** percentage of the injuries in each area out of all acute or overuse injuries among sports club members and non-members

The most common overuse injury location was the knee for sports club members (16.8% out of all overuse injuries in sports club members), this was the third most common location for non-members (11.1%). The second most common location for overuse injury was the hip, groin, gluteal and pelvis area for sports club members (11.1%) followed by the wrist and hand (10.0%). The most common location for an overuse injury for non-sports club members was the wrist or hand (Table 2).

The distributions of at least one injury by sports club membership, anatomic location, and sex are shown in Additional file 3: Table S3 for acute injuries and in Additional file 4: Table S4 for overuse injuries. Additional file 5: Table S5 shows the sex-adjusted details of at least one acute or overuse injury and its location in sports club members training more than three times compared to non-members.

### Injury types

The most common acute injuries in both groups were sprains 31.6% of sports club members reported a sprain during the past 12-month, compared to 13.5% of non-members (*P <* 0.001). Contusions/bruises other than the head were the second most common type of injury in both groups (sports club members 17.1% vs. non-members 7.6%, *P <* 0.001). The third most common acute injury type in sports club members was a muscle injury, and it was reported more often by members than non-members (13.5% vs. 4.7%). Out of non-members, 4.8% reported sustaining a fracture within the past year, this being the third most common type of acute injury for this group.

The most common overuse injury type was muscle injury among both sports club members and non-members (16.6% of sports club members and 10.1% of non-members reported at least one muscle injury, *P* < 0,001). Overuse injuries of the bone were more prevalent among sports club members than non-members (9.8% vs. 4.7%, *P <* 0.001). However, no statistically significant difference was seen in stress fractures between sports club members and non-members (2.9% vs. 1.6%, *P =* 0.075).

### Circumstances and causes of acute injuries

The most common setting for acute injuries in sports club members was organized training (25.4%) followed by matches or competitions (22.2%). Among sports club members, acute injuries in matches or competitions were more common in boys than girls (27.7% vs. 16.5%, *P <* 0.001). There was no difference between sports club members and non-members in acute injuries occurring during leisure time activities (16.0% vs. 17.1%) or school sports (7.4% vs. 7.0%). Among non-members, boys were more likely to report an acute injury during leisure time activities than girls (21.2%vs. 14.1%, *P =* 0.028). In both groups, the main cause for an acute injury was falling/stumbling (sports club members 16.8% vs. non-members 10.3%, *P <* 0.001). In sports club members, other common reasons for an acute injury were tackling (11.0% vs. 2.3%, *P <* 0.001 compared to non-members) and running (10.3% vs. 4.3%, *P <* 0.001).

### Training habits and injuries in sports club members

For those with training history for more than five years, training at least twice a week, the sex-adjusted OR for an acute injury was 1.38 compared to those who had trained actively for 0–4 years (*P =* 0.029; Table 3). Adolescents with weekly training for 7–14 h during the training season were 1.61 fold more likely to report an acute injury (*P =* 0.001) compared to adolescents who trained 3–6 h per week.

**Table 3:** Odds ratios (ORs) for at least one acute injury during the past twelve months in sports club members

						Sports club members						
	All (*n* = 1077)					Boys (*n* = 549)				Girls (*n* = 528)		
Training Characteristics	OR*	95% CI		*P* Value#		OR	95% CI	*P* Value^§^	OR	95% CI		*P* Value^¤^
Main sports												
Team and contact sports	1.0					1.0			1.0			
Individual sports	0.59	0.37–0.65		< 0.001		0.45	0.28–0.72	0.001	0.70	0.48–1.01		0.059
Starting training (age)												
4–7 years old	1.0					1.0			1.0			
8–11 years old	0.99	0.76–1.31		0.978		0.82	0.56–1.20	0.305	1.24	0.82–1.86		0.304
12–15 years old	0.60	0.42–0.85		0.004		0.59	0.36–0.97	0.037	0.65	0.39–1.07		0.090
Active training years at least 2 times/week												
0–4 years	1.0					1.0			1.0			
5–8 years	1.38	1.03–1.83		0.029		1.55	1.01–2.37	0.045	1.24	0.84–1.83		0.274
9–11 years	1.84	1.31–2.58		< 0.001		1.89	1.19–2.98	0.007	1.88	1.13–3.14		0.016
Training hours per week during training season												
3–6 h/week	1.0					1.0			1.0			
7–14 h/week	1.61	1.21–2.12		0.001		1.57	1.06–2.32	0.025	1.65	1.11–2.45		0.014
15 h or more/week	2.12	1.42–3.15		< 0.001		2.16	1.28–3.64	0.004	2.04	1.10–3.76		0.023
Training hours per week during competition season												
3–6 h/week	1.0					1.0			1.0			
7–14 h/week	1.55	1.18–2.06		0.002		1.39	0.94–2.04	0.097	1.77	1.17–2.67		0.006
15 h or more/week	1.78	1.20–2.63		0.004		1.67	0.99–2.81	0.055	1.91	1.05–3.48		0.033
The amount of competitions during last 12 months												
7–19 competitions	1.0					1.0			1.0			
20–39 competitions	1.34	0.93–1.92		0.118		1.48	0.89–2.47	0.132	1.29	0.77–2.15		0.331
40 competitions or more	1.55	1.05–2.28		0.028		2.02	1.21–3.37	0.007	0.92	0.48–1.77		0.809
Resting days per week during training season												
3 resting days/week	1.0					1.0			1.0			
2 resting days/week	1.13	0.79–1.59		0.507		1.25	0.77–2.02	0.371	1.00	0.61–1.67		0.977
1 resting day/week	1.35	0.95–1.93		0.098		1.54	0.94–2.53	0.088	1.18	0.70–1.96		0.538
0 resting day/week	1.62	0.61–4.26		0.333		1.30	0.36–4.78	0.688	2.16	0.49–9.53		0.310

^*^ OR = Odds Ratio, 95% CI = 95% Confidence Interval

^#^*P* Values in all sports club members derived from logistic regression adjusted for sex

^§^*P* Values in boys derived from logistic regression

¤ *P* Values in girls derived from logistic regression

Sports club members participating in 40 competitions or more per year were more likely to report an acute injury (*P =* 0.028) compared to those having 7–19 competitions a year. This was seen particularly among boys (*P =* 0.007) but not among girls (*P =* 0.809; Table 3). The mean amount of competitions during the past twelve months was higher among boys than girls (30.1 vs. 15.1, *P <* 0.001).

Having an overuse injury was 1.63 fold more likely (*P* = 0.001) in adolescents who trained 7–14 h weekly during the preceding training season compared to those training 3–6 h. Those adolescents participating in 40 competitions or more per year were significantly more likely to report an overuse injury in all adolescents (*P =* 0.038) compared to those participating in 7–19 competitions (Table 4).

**Table 4:** Odds ratios (ORs) for at least one overuse injury during the past twelve months in sports club members

				Sports club members					
	All (*n* = 1077)			Boys (*n* = 549)			Girls (*n* = 528)		
Training Characteristics	OR*	95% CI	*P* Value#	OR	95% CI	*P* Value ^§^	OR	95% CI	*P* Value¤
Main sports									
Team and contact sports	1.0			1.0			1.0		
Individual sports	0.88	0.66–1.18	0.401	0.99	0.62–1.58	0.981	0.81	0.56–1.19	0.288
Starting training (age)									
4–7 years old	1.0			1.0			1.0		
8–11 years old	0.93	0.70–1.23	0.595	0.73	0.49–1.09	0.123	1.14	0.75–1.72	0.549
12–15 years old	0.77	0.54–1.10	0.155	1.02	0.62–1.69	0.923	0.64	0.38–1.07	0.086
Active training years at least 2 times/week									
0–4 years	1.0			1.0			1.0		
5–8 years	1.33	0.99–1.79	0.056	1.17	0.75–1.82	0.488	1.49	1.00–2.21	0.048
9–11 years	1.02	0.72–1.46	0.898	1.03	0.64–1.67	0.894	0.94	0.55–1.63	0.835
Training hours per week during training season									
3–6 h/week	1.0			1.0			1.0		
7–14 h/week	1.63	1.21–2.18	0.001	1.91	1.24–2.94	0.003	1.42	0.95–2.11	0.088
15 h or more/week	1.66	1.11–2.50	0.014	2.14	1.24–3.71	0.007	1.23	0.66–2.30	0.513
Training hours per week during competition season									
3–6 h/week	1.0			1.0			1.0		
7–14 h/week	1.48	1.10–1.98	0.009	1.64	1.08–2.49	0.021	1.33	0.88–2.01	0.173
15 h or more/week	1.97	1.32–2.94	0.001	2.26	1.31–3.90	0.003	1.68	0.92–3.05	0.091
The amount of competitions during last 12 months									
7–19 competitions	1.0			1.0			1.0		
20–39 competitions	1.39	0.96–2.02	0.085	1.21	0.71–2.06	0.492	1.60	0.95–2.71	0.080
40 competitions or more	1.53	1.02–2.30	0.038	1.42	0.83–2.41	0.197	1.57	0.81–3.05	0.178
Resting days per week during training season									
3 resting days/week	1.0			1.0			1.0		
2 resting days/week	1.15	0.80–1.65	0.448	1.07	0.64–1.77	0.807	1.25	0.75–2.08	0.400
1 resting day/week	1.21	0.84–1.75	0.312	1.30	0.78–2.18	0.312	1.11	0.66–1.88	0.689
0 resting day/week	1.17	0.44–3.16	0.750	1.30	0.34–4.89	0.702	1.04	0.23–4.59	0.961

^*^ OR = Odds Ratio, 95% CI = 95% Confidence Interval

^#^*P* Values in all sports club members derived from logistic regression adjusted for sex

^§^*P* Values in boys derived from logistic regression

¤ *P* Values in girls derived from logistic regression

### Overall leisure-time physical activity and injuries by sport club membership

The distributions of leisure-time physical activity between sports club members and non-members is shown in Additional file 6: Table S6. We found that the probability of an acute injury for those who reported “physical activity for about 30 min per week” was 7.8 fold (*P* = 0.007) and for those who reported “7 h or more physical activity per week” it was 20.3 fold (*P* < 0.001) compared to those who reported “no physical activity”. When we included sports club participation in the model the sex-adjusted probability for reporting an acute injury for those non-members who reported “7 h or more physical activity per week” was 10.1 fold (*P* = 0.002) and for those who participated in sports clubs it was 2.4 fold (*P* < 0.001) (Additional file 7: Table S7).

The probability of reporting an overuse injury for those who reported “physical activity for about 30 min per week” was 3.7 fold (*P* = 0.045) compared to those not reporting any physical activity and the likeliness increased with increasing physical activity. For those who reported “7 h or more physical activity per week” it was 9.8 fold (*P* < 0.001) compared to those who reported “no physical activity”. When we included sports club participation in the model the sex-adjusted probability for reporting an overuse injury for those non-members who reported “7 h or more physical activity per week” was 6.5 fold higher (*P* = 0.002) and for those who participated in sports clubs it was 1.7 fold higher (*P* < 0.001) (Additional file 7: Table S7).

## Discussion

Sixty percent of sports club members and 30 % of non-members reported at least one acute or overuse injury during the past twelve months. The probability for sustaining an acute or overuse injury increased with the increasing number of training hours and competitions among sports club members. However, having less than three resting days per week did not increase an injury. Acute injuries were reported more often in contact and team sports than in individual sports among sports club members.

The sports club members and non-members formed a representative sample of the different regions of Finland and the sports club sample covered the 10 most popular sports in Finland. Both individual and team sports and summer and winter sports were equally represented [28]. Both groups should have similar accuracy in injury data and recall. The terms “acute injury” and “overuse injury” were defined in the questionnaire—as validated earlier [19, 25, 26] —to make the answers more reliable, and acute and overuse injuries were looked at separately.

One limitation of our study is the cross-sectional nature of this study. Also the self-reported questionnaire may cause recall bias and sports club members may remember their injury history better. However, the questionnaires used in these surveys were compiled from previously validated questions [19, 23–26]. The non-respondents were 9th grade students as were those who took part in the study, we do not have further details concerning the non-respondents. We believe that the risk for either participating or not participating is the same for both injured and non-injured adolescents. However, there may be a risk that injured athletes had withdrawn themselves from sports club activities because of an injury/injuries and would be non-members with an injury sustained in a sports club setting. Although, at the age of 15 only a few injuries are so severe that they cause withdrawal from sports participation. There may also be a risk that sports club members understood the terms an acute and an overuse injury more clearly than non-members, but the definitions were written clearly and their differences were underlined to minimize the risk of misunderstanding. In the self-reported questionnaire there is always a risk that adolescent is not aware of the name of the anatomic location that is injures, but this is the case in the both study groups, and thus, the results should be comparable. A further limitation is that training details were only inquired from the sports club member group, since reporting training hours may have a different meaning and be less valid among non-members. We also carried out an additional analysis of the occurrence of injuries in both sports club members and non-members using an identical question on leisure-time physical activity volume for both groups.

### Injury occurrence and profile

Different sports disciplines have their specific typical acute and overuse injuries. For optimal preventive measures, sports-specific injury profiles need to be known. Taking into account the intensity and type of sports performed by the Finnish adolescents, our results concerning athletic injuries in young people who are participating in sports are in line with previous findings [6, 29–32].

Leppänen et al. [6] studied overuse injuries among adolescent basketball and floorball players using a self-reported questionnaire. Among floorball players the knee was the most common site for an overuse injury, the same finding was made in our study. Bostrom et al. [29] studied seven different sports and found that nearly half of the young adults had been injured during the past 12 months, which is close to 44% of adolescents having sustained at least one acute injury in our study. In both studies boys were more often injured than girls. However, in the study by Bostrom et al. [29] an acute injury was defined by an absence time of one week from sports compared to one day in our study. In a study of high school athletes by Kahlenberg et al. [30] those with a higher total number of hours per year of sports participation had a higher risk of injury. In our study, both acute and overuse injury was significantly increased with 15 h or more of training per week.

In our study, the acute injury incidence rate was significantly higher in team and contact sports compared to individual sports, which is in accordance with previous studies [4, 5, 33, 34], and acute injuries occurred more frequently to boys than to girls. This reflects the sports type distribution among Finnish adolescents, which explains why this result deviates from the findings seen in specific sports with a similar degree of competitiveness, where girls have a higher incidence of injury than boys [35–37]. In our study individual sports that were more often performed by girls were orienteering, riding, dancing, gymnastics, and skating.

In our study, sports club members reported on average one overuse injury per year. This was more than in non-members, but the reported 0.7 overuse injuries per year among non-members also needs to be recognized. Our result is in line with that reported by Malisoux et al. [31], but again the risk for an overuse injury depends on the sport type [6].

The most common acutely injured location for both sports club members and non-members was the wrist and hand combined. The most common more specific location among sports club members were the ankle and foot, again in accordance with previous research [35, 38]. In our study, the most common site for an overuse injury was the knee for sports club members and the wrist and hand for non-members.

Earlier studies have reported that the knee is commonly injured in adolescents [39] and 18–35 year old adults [40] especially in ball games and running. The knee was second most common acute injury location in non-members whereas in sports club members it was the 5th most common injury location. Acute knee injuries may be relatively less common among sports-club members as a result of neuromuscular training targeted at injury prevention [41]. Acute knee injuries were reported by 12% of all participants, there was no difference between boys and girls but a greater number of acute knee injuries were reported by sports club members than non-members. Most acute knee injuries occurred in ball games and among girls in synchronized skating and gymnastics. In a previous study by Pasanen et al. [42] 18% of acute injuries in floorball were knee injuries, including 7 ACL injuries. In our study no ACL injuries were reported. None of the knee injuries reported in our study required more than three days away from normal training or competition, so we can assume they were minor injuries.

In our study 12% of overuse injuries were knee injuries, significantly more of these were reported by sports club members. In previous studies it has also been found that Osgood-Schlatter disease, patellofemoral pain, and other unspecified knee pain are common in adolescents participating in sports [43–45]. Leppänen et al. [46] found in previous study of overuse injuries in basketball and floorball players that 35% of overuse injuries were injuries of the knee, however the age group in this study was 12–20 years. In our study knee overuse injuries were most common in ball game players.

In line with previous research [35, 47, 48] the most common acute injury type in both groups was sprains followed by contusions or bruises. The most common type of overuse injury was muscle injury in both groups. This injury type includes, for example, chronic exertional compartment syndrome and delayed onset muscle soreness. However, it can be argued whether delayed onset muscle soreness (DOMS) should be classified as an overuse injury. DOMS is primarily caused by eccentric contractions that produce the greatest muscle tension [49]. Sports club members reported significantly more overuse injuries of the bone tissue than non-members. Included in these injuries may be, for example, Osgood-Schlatter disease. Stress fractures were not statistically significantly more common in sports club members. A potential cause for this may be that this type of injury may have caused withdrawal from sports club activities. Also only a small minority of sports club participants participated in intensive elite level training. Many earlier studies have found that participation in gymnastics, dance, and other high-impact activity carries a higher risk for stress fractures, especially in girls [50, 51].

### Injury circumstances and the need for prevention

The injury risk varies between different sports and other leisure time activities [52]. In accordance with earlier research [3, 35, 53] organized training was found to be the most common setting for acute injuries in sports club members, followed by matches or competitions. However, the injury risk is not always higher in organized sports. Monroe et al. [13] found that players taking part in organized soccer had a higher prevalence of injury compared to players of non-organized soccer. On the contrary, in basketball non-organized players had a higher prevalence of injury compared to players of organized basketball [13].

McQuillan and Campbell [34] reported that sports caused 32% of the injuries in 12–17-year-old adolescents. In non-members, most acute injuries occurred during leisure time. Abernethy and MacAuley [54] reported that 51% of accidental injuries occur during school sports, which was much more than in our study. In addition to formal physical education class, school sports included organized and casual sports performed at school. Although sports club members may have better balance because of their more versatile training methods, this does not cause a difference in the amount of injuries in school sports. This may be due to sports club members participating more intensively in school sports compared to non-members. The most frequently reported cause for an acute injury was falling or stumbling in both groups. Sports club members were more likely than non-members to sustain acute injuries in tackling situations. This can be explained by the high participation rate in team and contact sports [55].

Earlier studies have reported that greater sports participation is associated with an increased risk of injury both in adolescents and adults [19, 56]. In accordance with the results of our study, Richmond et al. [57] found that adolescents had a greater risk of a sports-related injury with increasing hours of play. In our study, training details were inquired only from sports club members, and we found that weekly training for over seven hours was associated with a higher amount of acute injuries in boys and girls. However, the amount of overuse injuries was significantly increased only among boys when training over seven hours per week. It has also been found that low levels of habitual physical activity increase the injury risk in children when exposure time is taken into account [58]. As reported earlier [4], the number of competitions is associated with the yearly incidence of injury, particularly in team games. In our study, boys reported having taken part in twice as many competitions during the preceding year than girls did.

We also surveyed the leisure-time physical activity from both groups. Only 30 min of weekly physical activity was found to significantly increase the likeliness of both the acute and overuse injuries. Injuries were further increased with increasing leisure-time physical activity in those completing 2 h or more of physical activity per week. This was seen more clearly in sports club members. This result is in agreement with previous research showing that more competitive forms of physical activity are associated with higher injury risk. [59, 60].

Effective preventive measures targeted at risk factors for both acute and overuse injuries are needed in both organized and non-organized sports in order to prevent injuries. Poor knee alignment and control during jump-landing may increase the risk of injury. However, these risk factors does not seem to change in young athletes by time by simply playing the sport. Improvement of knee stability needs specific neuromuscular exercises [39].

Physical activity programs that develop strength and proprioception have been shown to be effective in preventing acute and overuse sports injuries [61, 62]. Including this type of training as a part of physical education would also aid adolescents not participating in sports club activities to meet physical activity guidelines without injuries [63, 64].

## Conclusions

Physical activity provides a balance to the nowadays common sedentary lifestyle common among adolescents. Our study shows the quantity of the combined extent of acute and overuse injuries among adolescent sports club members compared to non-members. The sports type, such as team and contact sports, training, and competing habits influence this among adolescents. Injury prevention is needed, both in organized and non-organized sports.

## Additional files

Additional files 1: **Table S1.** Main sports reported by sports club members. (DOC 61 kb)

Additional files 2: **Table S2.** The main structured questions in the questionnaire. (DOC 43 kb)

Additional files 3: **Table S3.** Anatomical site of at least one acute injury in boys and girls among sports club members and non-members. (DOC 70 kb)

Additional files 4: **Table S4.** Anatomical site of at least one overuse injury in boys and girls among sports club members and non-members. (DOC 67 kb)

Additional files 5: **Table S5.** Comparison of at least one acute or overuse injury in sports club members (training three times or more per week during the training season) and non-members in the past twelve months. (DOC 62 kb)

Additional files 6: **Table S6.** Reported leisure-time physical activity volumes among sports club members and non-members. (DOC 50 kb)

Additional files 7: **Table S7.** Odds ratios for the occurrence of acute and overuse injury (at least one injury) by sports club participation and by volume of reported leisure-time physical activity derived from logistic regression analysis adjusted for sex. (DOC 62 kb)

## Abbreviations

ACL: Anterior cruciate ligament
CI: Confidence Interval
DOMS: Delayed onset muscle soreness
FHPSC: The Finnish Health Promoting Sports Club (FHPSC)
OR: Odds ratio
