# Supplementary material for: Acute and overuse injuries among sports club members and non-members: the Finnish Health Promoting Sports Club (FHPSC) study
Source: BMC Musculoskelet Disord. 2019 Jan 19;20:32. doi: 10.1186/s12891-019-2417-3 (PMC6339310; doi:10.1186/s12891-019-2417-3)
Supplement: Supplementary file 1 — Table S1. Main sports reported by sports club members. (DOC 61 kb) [file 12891_2019_2417_MOESM1_ESM.doc]

Supplementary table

**Table S1** Main sports reported by sports club members

|  |  |  |  |  |
| --- | --- | --- | --- | --- |
|  | All | Boys | Girls | *P* Value# |
|  | *n* (%) | *n* (%) | *n* (%) |  |
|  | 1,074* | 547 | 527 |  |
| **Team sports** | 564 (52.5)¤ | 405 (74.0) | 159 (30.2) | <0.001 |
| Soccer | 166 (15.5) | 110 (20.1) | 56 (10.6) | <0.001 |
| Ice-hockey | 146 (13.4) | 138 (25.2) | 8 (1.5) | <0.001 |
| Floorball | 137 (12.8) | 107 (19.6) | 30 (5.7) | <0.001 |
| Basketball | 63 (5.9) | 31 (5.7) | 32 (6.1) | 0.790 |
| Volleyball | 27 (2.5) | 3 (0.5) | 24 (4.6) | <0.001 |
| Water polo | 11 (1.0) | 6 (1.1) | 5 (0.9) | 0.811 |
| Finnish baseball | 5 (0.5) | 4 (0.7) | 1 (0.2) | 0.195 |
| American football | 4 (0.4) | 4 (0.7) | 0 (0.0) | 0.050 |
| Handball | 2 (0.2) | 1 (0.2) | 1 (0.2) | 0.979 |
| Ringette | 2 (0.2) | 0 (0.0) | 2 (0.4) | 0.150 |
| Bandy | 1 (0.1) | 1 (0.2) | 0 (0.0) | 0.327 |
| **Individual sports** | 304 (28.3) | 116 (21.2) | 188 (35.7) | <0.001 |
| Track and field | 74 (6.9) | 32 (5.9) | 42 (8.0) | 0.201 |
| Skiing sports | 57 (5.3) | 31 (5.7) | 26 (4.9) | 0.611 |
| Orienteering | 43 (4.0) | 13 (2.4) | 30 (5.7) | 0.008 |
| Riding | 42 (3.9) | 0 (0.0) | 42 (8.0) | <0.001 |
| Swimming | 36 (3.4) | 13 (2.4) | 23 (4.4) | 0.080 |
| Combat sports | 36 (3.4) | 20 (3.7) | 16 (3.0) | 0.585 |
| Racquet sports | 16 (1.5) | 7 (1.3) | 9 (1.7) | 0.568 |
| **Team and individual sports** | 175 (16.3) | 5 (0.9) | 170 (32.3) | <0.001 |
| Dancing, gymnastics | 121 (11.3) | 3 (0.5) | 118 (22.4) | <0.001 |
| Skating | 54 (5.0) | 2 (0.4) | 52 (9.9) | <0.001 |
| **Other sports** | 31 (2.9) | 21 (3.7) | 10 (1.9) | 0.065 |
|  | | | |  |

*Out of 1077 sports club members, three did not respond the question “what is your main sport?”

#*P* Values for statistical difference for the proportion of boys and girls among sports club members

^¤^Percentages: Sports club members with main sports for instance team sports compared to all sports club members (1074) who reported their main sports.

^$^Other sports included agility, fencing, parkour, ultimate, paddling, bowling, sailing, diving, climbing, triathlon, and floating.
