# Supplementary material for: Acute and overuse injuries among sports club members and non-members: the Finnish Health Promoting Sports Club (FHPSC) study
Source: BMC Musculoskelet Disord. 2019 Jan 19;20:32. doi: 10.1186/s12891-019-2417-3 (PMC6339310; doi:10.1186/s12891-019-2417-3)
Supplement: Supplementary file 2 — Table S2. The main structured questions in the questionnaire. (DOC 43 kb) [file 12891_2019_2417_MOESM2_ESM.doc]

Supplementary table

**Table S2** The main structured questions in the questionnaire

1. Do you participate in sports club activities?
2. What is your main sport?
3. At what age did you start to practice your sport?
4. For how many years have you participated in sports actively (at least twice a week)?
5. How long is your average total weekly practice time for all sports during the training season? ____hours/week, ____times/week
6. How long is your average total weekly practice time for all sports during the competition season? ____hours/week, _____times/week
7. How many competitions have you participated in on average over the past twelve months?
8. How many whole rest days per week do you take on average during the training season?
9. How many whole rest days per week do you take on average during the competition season?
10. Have you had an acute leisure time or sports injury in the past twelve months?
11. Where was the acute injury located? (34 different anatomical site alternatives given in the structured question)
12. What type of acute injury did you sustain? (11 different injury type alternatives given in the structured question)
13. In what situation did the acute injury occur? (19 different situation alternatives given in the structured question)
14. Where did the acute injury occur? (10 different place alternatives given in the structured question)
15. Have you had an overuse injury in the past twelve months?
16. Where was the overuse injury located? (26 different anatomical site alternatives given in the structured question)
17. In which tissue did the overuse injury occur? (muscle, tendon, bone, joint, ligament, nerve tissue)

Question on leisure-time physical activity

1. How many hours of exercise/physical activity (so that get short of breath and sweat) do you usually do per week in your leisure time (after school lessons)?
