# Supplementary material for: Acute and overuse injuries among sports club members and non-members: the Finnish Health Promoting Sports Club (FHPSC) study
Source: BMC Musculoskelet Disord. 2019 Jan 19;20:32. doi: 10.1186/s12891-019-2417-3 (PMC6339310; doi:10.1186/s12891-019-2417-3)
Supplement: Supplementary file 3 — Table S3. Anatomical site of at least one acute injury in boys and girls among sports club members and non-members. (DOC 70 kb) [file 12891_2019_2417_MOESM3_ESM.doc]

Supplementary table

**Table S3** Anatomical site of at least one acute injury in boys and girls among sports club members and non-members

|  | Sports club members | | |  | Non-members | | |  |  |
| --- | --- | --- | --- | --- | --- | --- | --- | --- | --- |
|  | All | Boys | Girls | *P* Value^*^ | All | Boys | Girls | *P* Value^*^ | *P* Value^#^ |
|  | *n* = 1,077 | *n* = 549 | *n* = 528 |  | *n* = 812 | *n* = 345 | *n* = 467 |  |  |
|  | *n* (%) | *n* (%) | *n* (%) |  | *n* (%) | *n* (%) | *n* (%) |  |  |
| Acute injury (yes) | 474 (44.0) | 256 (46.6) | 218 (41.3) | 0.077 | 161 (19.8) | 73 (21.2) | 88 (18.8) | 0.413 | <0.001 |
| Head | 47 (4.4) | 26 (4.7) | 21 (4.0) | 0.560 | 20 (2.5) | 11 (3.2) | 9 (1.9) | 0.264 | 0.432 |
| Face, teeth, eye area | 35 (3.2) | 22 (4.0) | 13 (2.5) | 0.166 | 14 (1.7) | 7 (2.0) | 7 (1.5) | 0.573 | 0.600 |
| Shoulder, upper arm, clavicle | 72 (6.7) | 45 (8.2) | 27 (5.1) | 0.058 | 24 (3.0) | 16 (4.6) | 8 (1.7) | 0.018 | 0.975 |
| Elbow, forearm | 33 (3.1) | 22 (4.0) | 12 (2.3) | 0.115 | 18 (2.2) | 10 (2.9) | 8 (1.7) | 0.268 | 0.112 |
| Wrist and hand | 150 (13.9) | 82 (14.9) | 68 (12.9) | 0.397 | 60 (7.4) | 29 (8.4) | 31 (6.6) | 0.377 | 0.271 |
| Neck, neck region | 42 (3.9) | 26 (4.7) | 16 (3.0) | 0.164 | 24 (3.0) | 11 (3.2) | 13 (2.8) | 0.744 | 0.037 |
| Upper back | 32 (3.0) | 21 (3.8) | 11 (2.1) | 0.102 | 18 (2.2) | 7 (2.0) | 11 (2.4) | 0.760 | 0.084 |
| Low back | 81 (7.5) | 50 (9.1) | 31 (5.9) | 0.062 | 38 (4.7) | 19 (5.5) | 19 (4.1) | 0.360 | 0.080 |
| Chest | 31 (2.9) | 22 (4.0) | 9 (1.7) | 0.028 | 18 (2.2) | 10 (2.9) | 8 (1.7) | 0.268 | 0.050 |
| Abdomen | 26 (2.4) | 16 (2.9) | 10 (1.9) | 0.287 | 17 (2.1) | 8 (2.3) | 9 (1.9) | 0.706 | 0.032 |
| Hip, groin, gluteals, pelvis | 95 (8.8) | 62 (11.3) | 33 (6.3) | 0.008 | 24 (3.0) | 14 (4.1) | 10 (2.1) | 0.122 | 0.177 |
| Thigh | 75 (7.0) | 49 (8.9) | 26 (4.9) | 0.016 | 21 (2.6) | 13 (3.8) | 8 (1.7) | 0.076 | 0.434 |
| Knee | 164 (15.2) | 90 (16.4) | 74 (14.0) | 0.351 | 71 (8.7) | 33 (9.6) | 38 (8.1) | 0.515 | 0.045 |
| Calf and shin | 53 (4.9) | 27 (4.9) | 26 (4.9) | 0.996 | 19 (2.3) | 9 (2.6) | 10 (2.1) | 0.671 | 0.956 |
| Ankle | 195 (18.1) | 98 (17.9) | 97 (18.4) | 0.853 | 55 (6.8) | 23 (6.7) | 32 (6.9) | 0.923 | 0.072 |
| Achilles tendon | 33 (3.1) | 22 (4.0) | 11 (2.1) | 0.076 | 15 (1.8) | 8 (2.3) | 7 (1.5) | 0.400 | 0.323 |
| Foot | 111 (10.3) | 57 (10.4) | 54 (10.2) | 0.940 | 36 (4.4) | 14 (4.1) | 22 (4.7) | 0.669 | 0.571 |
| ^*^ *P* Values for statistical difference for the proportion of boys and girls among sports club members and non-members | | | | | | |  |  |  |
| ^#^ *P* Values for all injury locations between sports club members and non-members derived from logistic regression analysis adjusted for sex | | | | | | | |  |  |
