# Supplementary material for: Acute and overuse injuries among sports club members and non-members: the Finnish Health Promoting Sports Club (FHPSC) study
Source: BMC Musculoskelet Disord. 2019 Jan 19;20:32. doi: 10.1186/s12891-019-2417-3 (PMC6339310; doi:10.1186/s12891-019-2417-3)
Supplement: Supplementary file 4 — Table S4. Anatomical site of at least one overuse injury in boys and girls among sports club members and non-members. (DOC 67 kb) [file 12891_2019_2417_MOESM4_ESM.doc]

Supplementary table

**Table S4** Anatomical site of at least one overuse injury in boys and girls among sports club members and non-members

|  | Sports club members | | |  | Non-members | | |  |  |
| --- | --- | --- | --- | --- | --- | --- | --- | --- | --- |
|  | All | Boys | Girls | *P* Value* | All | Boys | Girls | *P* Value^*^ | *P* Value# |
|  | *n* = 1,077 | *n* = 549 | *n* = 528 |  | *n* = 812 | *n* = 345 | *n* = 467 |  |  |
|  | *n* (%) | *n* (%) | *n* (%) |  | *n* (%) | *n* (%) | *n* (%) |  |  |
| Overuse injury (yes) | 378 (35.1) | 188 (34.2) | 190 (36.0) | 0.550 | 141 (17.4) | 51 (14.8) | 90 (19.3) | 0.095 | <0.001 |
| Shoulder, upper arm | 32 (3.0) | 18 (3.3) | 14 (2.7) | 0.556 | 21 (2.6) | 12 (3.5) | 9 (1.9) | 0.180 | 0.035 |
| Elbow, forearm | 21 (1.9) | 13 (2.4) | 8 (1.5) | 0.321 | 12 (1.5) | 8 (2.3) | 4 (0.9) | 0.093 | 0.175 |
| Wrist and hand | 62 (5.8) | 23 (4.2) | 39 (7.4) | 0.034 | 34 (4.2) | 13 (3.8) | 21 (4.5) | 0.623 | 0.138 |
| Neck, neck region | 30 (2.8) | 17 (3.1) | 13 (2.5) | 0.538 | 24 (3.0) | 10 (2.9) | 14 (3.0) | 0.936 | 0.005 |
| Upper back | 29 (2.7) | 20 (3.6) | 9 (1.7) | 0.056 | 20 (2.5) | 10 (2.9) | 10 (2.1) | 0.502 | 0.020 |
| Low back | 94 (8.7) | 50 (9.1) | 44 (8.3) | 0.404 | 41 (5.0) | 18 (5.2) | 23 (4.9) | 0.858 | 0.450 |
| Chest | 18 (1.7) | 12 (2.2) | 6 (1.1) | 0.187 | 12 (1.5) | 9 (2.6) | 3 (0.6) | 0.024 | 0.070 |
| Abdomen | 15 (1.4) | 10 (1.8) | 5 (0.9) | 0.227 | 13 (1.6) | 7 (2.0) | 6 (1.3) | 0.411 | 0.019 |
| Hip, groin, gluteals, pelvis | 74 (6.9) | 39 (7.1) | 35 (6.6) | 0.774 | 30 (3.7) | 13 (3.8) | 17 (3.6) | 0.927 | 0.844 |
| Thigh | 57 (5.3) | 33 (6.0) | 24 (4.5) | 0.308 | 25 (3.1) | 12 (3.5) | 13 (2.8) | 0.583 | 0.473 |
| Knee | 166 (15.4) | 89 (16.2) | 77 (14.6) | 0.527 | 60 (7.4) | 21 (6.1) | 39 (8.4) | 0.257 | 0.592 |
| Calf and shin | 67 (6.2) | 27 (4.9) | 40 (7.6) | 0.090 | 25 (3.1) | 12 (3.5) | 13 (2.8) | 0.583 | 0.690 |
| Ankle | 66 (6.1) | 29 (5.3) | 37 (7.0) | 0.216 | 43 (5.3) | 17 (4.9) | 26 (5.6) | 0.703 | 0.005 |
| Achilles tendon | 28 (2.6) | 19 (3.5) | 9 (1.7) | 0.078 | 17 (2.1) | 9 (2.6) | 8 (1.7) | 0.388 | 0.079 |
| Foot | 61 (5.7) | 31 (5.6) | 30 (5.7) | 0.981 | 36 (4.4) | 16 (4.6) | 20 (4.3) | 0.816 | 0.026 |
| **P* Values for statistical difference for the proportion of boys and girls among sports club members and non-members | | | | | | | |  |  |
| ^#^ *P* Values for statistical difference for all injury locations between sports club members and non-members derived from logistic regression adjusted for sex | | | | | | | |  |  |
