# Supplementary material for: Acute and overuse injuries among sports club members and non-members: the Finnish Health Promoting Sports Club (FHPSC) study
Source: BMC Musculoskelet Disord. 2019 Jan 19;20:32. doi: 10.1186/s12891-019-2417-3 (PMC6339310; doi:10.1186/s12891-019-2417-3)
Supplement: Supplementary file 5 — Table S5. Comparison of at least one acute or overuse injury in sports club members (training three times or more per week during the training season) and non-members in the past twelve months. (DOC 62 kb) [file 12891_2019_2417_MOESM5_ESM.doc]

Supplementary table

***Table S5*** *Comparison of at least one acute or overuse injury in sports club members (training three times or more per week during the training season) and non-members in the past twelve months*

|  | **At least one acute injury** | |  | **At least one overuse injury** | |  |
| --- | --- | --- | --- | --- | --- | --- |
|  | Sports club members  Training 3 times /week or more during training season | Non-members |  | Sports club members  Training 3 times /week or more during training season | Non-members |  |
|  | *n* = 910 | *n* = 812 |  | *n* = 910 | *n* = 812 |  |
| Anatomical site of injury | *n* (%) | *n* (%) | *P* Value* | *n* (%) | *n* (%) | *P* Value* |
| Acute injury (yes) | 426 (46.8) | 161 (19.8) | <0.001 |  |  |  |
| Overuse injury (yes) |  |  |  | 335 (36.8) | 141 (17.4) | <0.001 |
| Head | 43 (4.7) | 20 (2.5) | 0.475 |  |  |  |
| Face, teeth, eye area | 30 (3.3) | 14 (1.7) | 0.527 |  |  |  |
| Shoulder, upper arm, clavicle | 66 (7.3) | 24 (3.0) | 0.932 | 28 (3.1) | 21 (2.6) | 0.030 |
| Elbow, forearm | 31 (3.4) | 18 (2.2) | 0.130 | 17 (1.9) | 21 (2.6) | 0.095 |
| Wrist and hand | 132 (14.5) | 60 (7.4) | 0.214 | 51 (5.6) | 34 (4.2) | 0.076 |
| Neck, neck region | 35 (3.8) | 24 (3.0) | 0.021 | 24 (2.6) | 24 (3.0) | 0.002 |
| Upper back | 29 (3.2) | 18 (2.2) | 0.098 | 24 (2.6) | 20 (2.5) | 0.011 |
| Low back | 73 (8.0) | 38 (4.7) | 0.086 | 82 (9.0) | 41 (5.0) | 0.343 |
| Chest | 28 (3.1) | 18 (2.2) | 0.054 | 15 (1.6) | 12 (1.5) | 0.045 |
| Abdomen | 24 (2.6) | 17 (2.1) | 0.047 | 13 (1.4) | 13 (1.6) | 0.018 |
| Hip, groin, gluteals, pelvis | 87 (9.6) | 24 (3.0) | 0.154 | 67 (7.4) | 30 (3.7) | 0.949 |
| Thigh | 70 (7.7) | 21 (2.6) | 0.358 | 51 (5.6) | 25 (3.1) | 0.452 |
| Knee | 150 (16.5) | 71 (8.7) | 0.063 | 147 (16.2) | 60 (7.4) | 0.628 |
| Calf and shin | 44 (4.8) | 19 (2.3) | 0.774 | 62 (6.8) | 25 (3.1) | 0.538 |
| Ankle | 177 (19.5) | 55 (6.8) | 0.060 | 56 (6.2) | 43 (5.3) | 0.003 |
| Achilles tendon | 30 (3.3) | 15 (1.8) | 0.345 | 24 (2.6) | 17 (2.1) | 0.071 |
| Foot | 93 (10.2) | 36 (4.4) | 0.896 | 49 (5.4) | 36 (4.4) | 0.008 |
| ^*^ *P* Values between sports club members (training 3 times / week or more during training season) and non-members derived from logistic regression adjusted for sex | | | | | | |
