# Supplementary material for: Acute and overuse injuries among sports club members and non-members: the Finnish Health Promoting Sports Club (FHPSC) study
Source: BMC Musculoskelet Disord. 2019 Jan 19;20:32. doi: 10.1186/s12891-019-2417-3 (PMC6339310; doi:10.1186/s12891-019-2417-3)
Supplement: Supplementary file 6 — Table S6. Reported leisure-time physical activity volumes among sports club members and non-members. (DOC 50 kb) [file 12891_2019_2417_MOESM6_ESM.doc]

Supplementary table

**Table S6** Reported leisure-time physical activity volumes among sports club members and non-members

|  |  |  |  |  |
| --- | --- | --- | --- | --- |
|  |  | Sports club members | Non-members |  |
|  |  | *n* = 1,033 | *n* = 764 |  |
|  |  | n (%) | n (%) | *P* Value* |
| How many hours of exercise/physical activity (to get out of breath and sweat) do you usually do per week in your leisure time (after school lessons)? | |  |  | < 0.001 |
|  | Not at all | 5 (0.5) | 43 (5.6) |  |
|  | About half an hour | 6 (0.6) | 85 (11.1) |  |
|  | About an hour | 37 (3.6) | 174 (22.8) |  |
|  | About 2 to 3 hours | 139 (13.5) | 247 (32.3) |  |
|  | About 4 to 6 hours | 311 (30.1) | 169 (22.1) |  |
|  | 7 hours or more | 533 (51.6) | 45 (5.9) |  |
|  | Empty | 2 (0.2) | 1(0.1) |  |
|  |  |  |  |  |

^*^ *P* Values between sports club members and non-members derived from logistic regression adjusted for sex
