# Supplementary material for: Acute and overuse injuries among sports club members and non-members: the Finnish Health Promoting Sports Club (FHPSC) study
Source: BMC Musculoskelet Disord. 2019 Jan 19;20:32. doi: 10.1186/s12891-019-2417-3 (PMC6339310; doi:10.1186/s12891-019-2417-3)
Supplement: Supplementary file 7 — Table S7. Odds ratios for the occurrence of acute and overuse injury (at least one injury) by sports club participation and by volume of reported leisure-time physical activity derived from logistic regression analysis adjusted for sex. (DOC 62 kb) [file 12891_2019_2417_MOESM7_ESM.doc]

Supplementary table

**Table S7** Odds ratios for the occurrence of acute and overuse injury (at least one injury) by sports club participation and by volume of reported leisure-time physical activity derived from logistic regression analysis adjusted for sex

|  |  |  |  |  |  |
| --- | --- | --- | --- | --- | --- |
|  | | **Injuries during past twelve months** | | | |
|  | | **OR*** | **95% CI** | | ***P* Value^#^** |
| ***Acute injury (at least one injury during a year)*** | |  |  | |  |
| Physical activity (hours/week) | |  |  | |  |
|  | Not at all | 1.0 |  | |  |
|  | About half an hour | 8.4 | 1.88-37.61 | | 0.005 |
|  | About an hour | 6.1 | 1.41-26.11 | | 0.015 |
|  | About 2 to 3 hours | 6.4 | 1.52-27.16 | | 0.011 |
|  | About 4 to 6 hours | 7.5 | 1.79-31.82 | | 0.006 |
|  | 7 hours or more | 10.1 | 2.40-42.89 | | 0.002 |
|  |  |  |  | |  |
| Sports club member or not | |  |  | |  |
|  | Non-member | 1.0 |  | |  |
|  | Sports club member | 2.4 | 1.85-3.14 | | <0.001 |
|  |  |  |  | |  |
| ***Overuse injury (at least one injury during a year)*** | |  |  |  |  |
| Physical activity (hours/week) | |  |  |  |  |
|  | Not at all | 1.0 |  | |  |
|  | About half an hour | 3.8 | 1.05-13.53 | | 0.042 |
|  | About an hour | 2.1 | 0.62-7.32 | | 0.234 |
|  | About 2 to 3 hours | 3.3 | 0.99-10.90 | | 0.052 |
|  | About 4 to 6 hours | 4.7 | 1.41-15.44 | | 0.012 |
|  | 7 hours or more | 6.5 | 1.96-21.67 | | 0.002 |
|  |  |  |  |  |  |
| Sports club member or not | |  |  |  |  |
|  | Non-member | 1.0 |  | |  |
|  | Sports club member | 1.7 | 1.31-2.28 | | <0.001 |

^*^OR = Odds Ratio, 95% CI = 95% Confidence Interval

^#^ *P* Values between sports club members and non-members derived from logistic regression adjusted for sex
